# Review of ”Bioinformatics for vaccinology“ edited by Darren R. Flower

**DOI:** 10.1186/1743-422X-6-85

**Published:** 2009-06-24

**Authors:** Dae-Won Kim, Hong-Seog Park

**Affiliations:** 1 Genome Research Center, Korea Research Institute of Bioscience and Biotechnology, Daejeon 305-806, Korea

## Abstract

Book review of "Bioinformatics for vaccinology" edited by Darren R. Flower.

## Book review

The study of vaccines has captured the attention of the biomedical and public communities for the past hundred years, largely because vaccines play a key role in affecting lifespan and well being, as well as economics. A positive trend in the field of vaccinology has been inspired by recent major breakthroughs in the fields of molecular biology, physiology, genomics, proteomics, and computers that promise a bright future for the prevention of infectious diseases and allergies. This book focuses on a fundamental understanding of the primary role of vaccines from a historical perspective, the immune system in molecular cell biology and computational approaches that show the link between experiment and theory.

The major portion of the book is divided into seven chapters. Chapter one provides a historical background of vaccination that acts as a framework for the remainder of the book. Overall, this chapter provides a comprehensive overview of several interesting subjects based on effective and efficient efforts at developing vaccines. Chapter two focuses on the introductory need and opportunity for vaccines, followed by the role of unprecedented phenomenon, such as rapid changes in economic status, climate and infectious disease. Chapter three describes how a rigorous biological perspective of the molecular immune system is a fundamental requirement for the development of computational algorithms to explain how vaccines work. It further provides a discussion of how informatics approaches can give a glimpse of vaccine discovery. Chapter four contains an introduction into '-omics' and reviews the most important biological sequence databases, such as the host databases, immunological databases, pathogen databases, and T cell and B cell databases. Chapter five describes the computational challenges in predicting T cell and B cell epitopes and in allergen discovery. Moreover, it provides a description of computational algorithms, including artificial neural networks (ANN), hidden Markov models (HMM), support vector machines (SVM), and introduces statistical models to assess prediction accuracy. Chapter six presents protein structural approaches for experimental screening using 3D-QSAR or for virtual screening (VS). Chapter seven addresses the importance and potential of computational vaccinology in the post-genomic era.

This book has several strong points. Although there are many textbooks that deal with vaccinology, few attempts have been made to bring together descriptions of vaccines in history, basic bioinformatics, various computational solutions and challenges in vaccinology, detailed experimental methodologies, and cutting-edge technologies. The book is aimed at researchers with a working knowledge of disease, the nature of vaccines, computational approaches and system biology. The lesser focus on basic concepts and theories may make this book undesirable as a textbook for beginners or undergraduate students in vaccinology. However, the book contains a large amount of information on computation and experimental techniques that would be highly useful in the study of vaccines and in the pharmaceutical industry or for researchers who intend to prepare and use vaccines in their research. This book may well serve as a first line of reference for all biologists and computer scientists.

One of the major goals of the incorporation of bioinformatics into vaccinology is to aid in the design of vaccines and to apply informatics approaches to the development of novel therapeutics and vaccine candidates. This book provides details of basic vaccinology and its application, beginning with a consideration of computational approaches. This presentation should provide readers of this book the opportunity to absorb the information about the biological immune system, the mathematical methods and the immunoinformatics. Overall, this textbook would be an excellent addition to the bookshelf of most scientists who encounter vaccinology in the drug discovery and development processes.

## Competing interests

The authors declare that they have no competing interests.

## Authors' contributions

DK: conception, drafting and final approval of the manuscript. HP: drafting and final approval of the manuscript.

